# Emotional, behavioural problems and cigarette smoking in adolescence: findings of a Greek cross-sectional study

**DOI:** 10.1186/1471-2458-10-57

**Published:** 2010-02-03

**Authors:** George Giannakopoulos, Chara Tzavara, Christine Dimitrakaki, Gerasimos Kolaitis, Vasiliki Rotsika, Yannis Tountas

**Affiliations:** 1 Centre for Health Services Research, Department of Hygiene, Epidemiology and Medical Statistics, Medical School, University of Athens, Athens, Greece; 2 Department of Child and Adolescent Psychiatry, Medical School, University of Athens, "Agia Sophia" Children's Hospital, Athens, Greece; 3 Community Mental Health Centre Byron-Kesariani, Department of Psychiatry, Medical School, University of Athens, Athens, Greece

## Abstract

**Background:**

Although several studies have reported findings concerning the association between smoking and emotional/behavioural problems, little research has investigated this association after controlling for confounding factors which have been found to be significantly correlated with both cigarette smoking and emotional/behavioural problems and may have a strong effect on the relationship between adolescents' mental health and smoking. The present study attempted to assess the association between adolescents' smoking status and their emotional/behavioural problems after controlling for a number of possible confounders (i.e. age, gender, parental smoking status, exposure to family smoking, family socioeconomic status, adolescents' leisure time) in a Greek nation-wide school-based sample.

**Methods:**

Participants completed a questionnaire which retrieved information about age, gender, family socioeconomic status, smoking status, parental smoking, adolescents' leisure time and emotional/behavioural problems. Data were modelled using multiple logistic regression analysis with adolescents' smoking status as the dependent variable.

**Results:**

A total of 1194 (i.e. 63% response rate) of self-reported questionnaires (40.1% boys, 59.9% girls; 12-18 years old) were returned. Data from 1030 participants with full data were analyzed. Cigarette smoking was strongly associated with higher levels of emotional/behavioural problems (p < 0.001) and the association was not moderated (OR = 1.13, 95% CI: 1.08-1.18) after controlling for the effects of other covariates. Emotional symptoms, conduct problems and hyperactivity/inattention were all significantly associated with adolescents' current smoking.

**Conclusions:**

This study supports the association between smoking and emotional/behavioural problems among adolescents. Addressing adolescents' needs regarding their emotional/behavioural health could be helpful in the development of effective anti-smoking strategies in school environment and elsewhere.

## Background

Cigarette smoking is a leading cause of morbidity and premature death in European countries with the majority (80-90%) of adult smokers beginning to smoke before 18 years of age [1]. In Greece, the prevalence of smoking in middle school adolescents is high. According to the Global Youth Tobacco Survey which was implemented during the academic year 2004-2005, about one-third of the students (aged 13-15 years) reported that they had tried tobacco in the past, while 16.20% reported being current users of tobacco products [2]. Another study [3] on alcohol and other drug use among students in 35 European countries has reported that lifetime smoking among the Greek students is below European average (50% vs. 66%), and the 30-day prevalence of smoking has the same tendency (28% compared with 35%). However, smoking in Greek adolescents constitutes an increasing problem in the absence of planned combined efforts through anti-smoking policies and tobacco control interventions [4]. The thorough investigation of the psychosocial context in which adolescent smoking appears is an essential process in developing well-designed smoking prevention interventions.

A number of epidemiological studies have examined the association of cigarette smoking with psychiatric disorders in adolescence. Conduct disorders, attention-deficit/hyperactivity disorder (ADHD) and clinical levels of aggression have been consistently related to adolescents' regular smoking [5-21]. A large Chinese study in a sample of 1360 adolescents has found that externalizing problems were significantly associated with ever smoking (OR = 1.60, 95% CI: 1.00-2.60) after adjustment for sociodemographic covariates and life stress [5]. Similarly, a Dutch population-based study among 5938 adolescents [22] has reported that adolescents with depressive feelings were more likely to report lifetime smoking (OR = 1.73, 95% CI: 1.46-2.05), and more likely to report regular smoking (OR = 2.06, 95% CI: 1.55-2.74) than those without depressive feelings. Significant effects were also found for age and education. However, the findings about internalizing disorders, such as depression and anxiety, are somewhat contradictory with some studies indicating a significant relationship between these disorders and smoking [5,23-25], while others did not manage to confirm a significant association [26-29]. Moreover, most of the reported studies have not examined the association of emotional/behavioural problems and tobacco use after controlling for confounding factors beyond age, gender and education, such as family socioeconomic status, parental smoking and adolescents' leisure time which have been found to be significantly correlated with both cigarette smoking and emotional/behavioural problems and may have a strong effect on the relationship between adolescents' mental health and smoking [30-33].

The present study was an effort to extend previous research through assessing smoking status, emotional/behavioural problems, and a number of other variables (i.e. adolescents' age and gender, family socioeconomic status, parental smoking, and adolescents' leisure time) in a Greek nation-wide school-based sample of adolescents. Given the relatively few previous studies on this issue, the present large survey attempted to contribute to a broader insight into the relationship between cigarette smoking and emotional/behavioural problems.

## Methods

### Participants and procedure

This study was conducted in the year 2003 within the framework of the European project 'Screening and Promotion for Health-Related Quality of Life (HRQoL) in Children and Adolescents: A European Public Health Perspective' [34]. The school sampling in Greece was random, multi-staged and performed to take into account distribution of the target population by age and administrative school region. The target population was adolescents aged 12 to 18. A sample size of 1800 adolescents was considered necessary to detect a minimally important difference of half a standard deviation (SD) in HRQoL scores within each age strata between children with and without special healthcare needs or a chronic condition. A response rate of approximately 70% was expected, so the initial sample size was set at 2400 children and adolescents. In Greece, ages 12 to 18 correspond to six secondary school grades. Approximately 400 students were included from each of the 6 age groups/grades in order to reach the original target of 2400 adolescents. For example, the total number of students in Greece attending the first grade of the secondary school is 119055. If an administrative region had a total number of 2174 students attending the first grade of the secondary school, then eight students were randomly recruited from a school in that region ((2174 × 400)/119055 = 7.60 students). Each age group/grade had been calculating accordingly, for each sector. Schools in each sector were randomly selected by a computer program and students of each selected school were selected randomly from classroom name lists. Inclusion criteria were adequate reading skills. A sample of 1900 adolescents (12 to 18 year olds) was recruited. Students were asked to complete the questionnaire at home after providing written informed consent. Ethical approval was attained from the National Ministry of Education.

### Measures

#### Adolescent and parental smoking status

Adolescents' smoking was assessed by questioning the participants "How often do you currently smoke cigarettes (or tobacco)?" Answer categories ranged from never to everyday. Parental smoking was assessed by questioning adolescents "Does your father smoke?" and "Does your mother smoke?" Answer categories were: yes/no. Additionally, participants were asked if any family member smoked in the place where adolescents did their homework or spent their free time. Answer categories were: yes/no.

#### Family socioeconomic status

To assess SES, the Family Affluence Scale [FAS;[35]] was used, addressing issues of family car ownership, having their own unshared room, the number of computers at home and time the adolescent spent on holiday in the past 12 months.The FAS was collected from adolescents in seven categories (from 0 the lowest, to 7 the highest FAS category) and was re-coded into three groups in the analysis (low FAS level (0-3), intermediate (4-5) and high FAS level (6-7)). The psychometric properties of the FAS are acceptable and support its use as a self-reported adolescents' measure [36].

#### Adolescents' leisure time

Adolescents' leisure time was measured using the dimension 'autonomy' of KIDSCREEN-52, a generic self-reported questionnaire with good psychometric properties [37]. This specific dimension was selected since previous literature has shown that leisure time is significantly associated with adolescents' smoking status [31]. This dimension is intended to explore the opportunity given to adolescents to create their social and leisure time. In particular, the extent to which adolescents feel able to shape their own life as well as being able to make decisions about day to day activities is considered. The dimension also examines if adolescents feel sufficiently provided with opportunities to participate in social activities particularly in leisure activities and pastimes. It consists of 5 items, with formats using a 5-point Likert response scale and the recall period being 1 week. Rasch scores are computed and transformed into T-values with a mean of 50 and a standard deviation of 10; higher scores indicate better physical well-being. The internal consistency coefficient for the autonomy score was 0.84 in the present sample.

#### Adolescents' emotional/behavioural problems

To assess adolescents' emotional/behavioural problems, the Strengths and Difficulties Questionnaire (SDQ) [38] was used. The SDQ contains 25 items (small sentences), categorized into five scales of five items each: hyperactivity/inattention, emotional symptoms, conduct problems, peer problems and prosocial behaviour. Responses to each of the 25 items consisted of three options: not true, somewhat true, or certainly true. For all scales the items that are worded negatively are assigned scores of 2 for certainly true, 1 for somewhat true, and 0 for not true. All but the last scale can be summed up to a total difficulties score ranging from 0 to 40. The version for youths was used in the present study. In order to combat inherent weaknesses of cross-cultural adaptation (e.g., semantic and scale equivalence) the research team in the present study followed a standardized translation methodology according to international cross-cultural translation guidelines [39]. The internal consistency coefficient for the total difficulties score was 0.77. Cronbach's alphas for the prosocial behaviour, emotional symptoms and hyperactivity-inattention were 0.72, 0.73 and 0.63, respectively. The lowest alpha was found on the peer problems (0.50) and conduct problems scale (0.56) in the present sample [40].

### Statistical analysis

Analyses were conducted concerning full data without missing values. Missing data counted less than 5% for each variable, but concerning all variables 1030 participants out of 1194 with full data were analyzed and reported. Continuous variables are presented with mean and standard deviation, while discreet variables are presented with absolute and relative frequencies. For the comparisons of proportions chi-square tests were used. Student's t-tests were computed for the comparison of mean values. Differences on SDQ scales according to smoking status were determined by the use of multivariate analysis of variance (MANOVA). Data were modelled using multiple logistic regression analysis with adolescents' smoking status as the dependent variable. The regression equation included terms for gender, age, KIDSCREEN-52 'autonomy' dimension, SDQ total difficulties scale, FAS, father's and mother's smoking status and family member's smoking in the place that adolescents do their homework or spend their free time. Adjusted odds ratios with 95% confidence intervals were computed from the results of the logistic regression analyses. Model diagnostics were evaluated using the Hosmer and Lemeshow statistic. All *p*-values reported are two-tailed. Statistical significance was set at 0.05 and analyses were conducted using SPSS statistical software (version 13.0).

## Results

A total of 1194 (i.e. 63% response rate) self-reported questionnaires (40.07% boys) were returned. Sample characteristics of the 1030 participants with full data are presented in Table 1. Of adolescents, 39.20% were male, while 60.8% were female. Close to half the sample had an intermediate level of family affluence (45.60%) and 32.40% were aged more than 15 years. More than one third of mothers were smokers (39.10%), while the correspondence proportion for fathers was higher and equal to 53.40%. Almost 36% of adolescents reported that there was at least one member in the family who smoked in the place where they did their homework or spent their free time. Approximately a tenth of adolescents reported smoking currently (1.70% less than once a week, 3.10% at least once a week and 5.40% every day).

**Table 1:** Sample characteristics

	*n*	%
**Age (years)**		
12-15	696	67.60
16-18	334	32.40
**Gender**		
Girls	626	60.80
Boys	404	39.20
**Family socioeconomic status**		
Low	376	36.50
Medium	470	45.60
High	184	17.90
**Mother smoker**		
No	627	60.90
Yes	403	39.10
**Father smoker**		
No	480	46.60
Yes	550	53.40
**Exposure to family members' smoking**		
No	654	63.50
Yes	376	36.50
**Smoking status**		
Never	925	89.80
Less than once a week	17	1.70
At least once a week	32	3.10
Every day	56	5.40

In univariate analysis (Table 2) it was found that the proportion of smokers was greater in adolescents aged 16 to 18 years (23.70%) compared to those aged 12 to 15 years (3.70%). No gender differences were found, but the proportion of adolescent smokers was greater in those whose mothers (14.40% vs. 7.50%) or fathers (13.10 vs. 6.90%) were smokers. Furthermore, smoking was more frequent in those whose family members smoked in the place where they did their homework or spent their free time. Additionally, the proportion of adolescent smokers was greater for those belonging to low FAS level (15.70%) compared to those belonging to middle (7.20%) or high (6.50%) level. Multivariate analysis of variance (Table 3) showed that smoking was associated with greater values on all SDQ scales except for prosocial behavior. The mean value for the SDQ total difficulties score among adolescents was 11.00 (SD = 2.50) for non-smokers and 14.60 (SD = 6.00) for smokers (*p *< 0.001).

**Table 2:** Sample characteristics by smoking status

	Smoking status				
	No		Yes		
		
	*n*	%	*n*	%	P χ^2 ^test
**Age (years)**					
12-15	670	96.30	26	3.70	<0.001
16-18	255	76.30	79	23.70	
**Gender**					
Girls	557	89.00	69	11.00	0.274
Boys	368	91.10	36	8.90	
**Family socioeconomic status**					
Low	317	84.30	59	15.70	<0.001
Medium	436	92.80	34	7.20	
High	172	93.50	12	6.50	
**Mother smoker**					
No	580	92.50	47	7.50	<0.001
Yes	345	85.60	58	14.40	
**Father smoker**					
No	447	93.10	33	6.90	0.001
Yes	478	86.90	72	13.10	
**Exposure to family members' smoking**					
No	601	91.90	53	8.10	0.003
Yes	324	86.20	52	13.80	
**Leisure time, mean (SD)**	58.60(23.40)		57.70(23.60)		0.720*

*Student's t-test

**Table 3:** Means and standard deviations of the Strengths and Difficulties Questionnaire (SDQ) scales by smoking status

	Smoking status					
	No		Yes			
			
	Mean	SD	Mean	SD	F (df: 1, 1026)	P*
**Emotional symptoms**	2.90	2.10	3.70	2.40	12.85	<0.001
**Conduct problems**	2.90	1.50	3.70	1.60	31.06	<0.001
**Hyperactivity-inattention**	3.40	2.10	4.80	2.30	33.58	<0.001
**Peer problems**	1.80	1.70	2.10	1.60	4.38	0.037
**Prosocial behaviour**	8.10	1.80	7.80	2.00	2.20	0.138

*multivariate analysis of variance (age and gender were used as covariates)

Multivariate analysis (Table 4) revealed that age, SDQ total difficulties score, FAS level, and parental smoking were independently associated with adolescents' smoking. Adolescents aged more than fifteen years had 8.56 times greater odds (95% CI: 4.89-14.97) for smoking compared to those aged from 12 to 15 years. Also, the odds for smoking was significantly lower for adolescents belonging to middle (OR = 0.43, 95% CI: 0.26-0.71) or high (OR = 0.46, 95% CI: 0.23-0.94) FAS level compared to those belonging to low level. The risk for smoking was almost twofold (OR = 1.94, 95% CI: 0.65-1.88) for those whose father was a smoker and 2.56 times greater (OR = 2.56, 95% CI: 1.49-4.39) for those whose mother was a smoker (Table 4). Greater scores on SDQ total scores were associated with greater likelihood for smoking with odds ratio equal to 1.13 (95% CI: 1.08-1.18).

**Table 4:** Results from multiple logistic regression that evaluated demographic/family factors and emotional/behavioural problems in relation to the presence of smoking habits

Explanatory variables	OR(95% CI)
**Age (years)**	
12-15	1.00‡
16-18	8.56(4.89-14.97)
**Gender**	
girl	1.00
boy	0.90(0.52-1.54)
**Family socioeconomic status**	
low 0-3	1.00
medium 4-5	0.43(0.26-0.71)
high 6-7	0.46(0.23-0.94)
**Mother smoker**	
No	1.00
Yes	2.56(1.49-4.39)
**Father smoker**	
No	1.00
Yes	1.94(1.11-3.4)
**Exposure to family members' smoking**	
No	1.00
Yes	1.11(0.65-1.88)
**Leisure time (for one unit increase)**	1.01(0.99-1.02)
**SDQ total difficulties (for one unit increase)**	1.13(1.08-1.18)

Abbreviations: OR = Odds Ratio, CI = 95% Confidence Interval, SDQ = Strengths and Difficulties

Questionnaire ‡ indicates reference category

Finally, multiple logistic regression analysis (Table 5) showed that all SDQ scales except for peer problems and prosocial behavior scale were associated with smoking also after adjustment for several selected covariates. The odds ratios for emotional symptoms, conduct problems and hyperactivity-inattention scales were 1.14, 1.36 and 1.28 (95% CI: 1.02-1.26, 1.18-1.56, and 1.15-1.43) respectively.

**Table 5:** Results from multiple logistic regression that evaluated emotional/behavioural problems in relation to the presence of smoking habits

	OR(95% CI)*	
Emotional symptoms	1.14(1.02-1.26)	
Conduct problems	1.36(1.18-1.56)	
Hyperactivity-inattention	1.28(1.15-1.43)	
Peer problems	1.09(0.95-1.25)	
Prosocial behaviour	0.94(0.84-1.05)	

*Adjusted for age, gender, leisure time, family socioeconomic status, parents' smoking habits, and exposure to family members' smoking

## Discussion

The present study investigated cigarette smoking among a Greek nation-wide school-based sample of adolescents and the relationship between cigarette smoking status and adolescents' emotional/behavioural problems while considering the potential confounding effects of other important factors (i.e. age, gender, socioeconomic status, parental smoking, leisure time) in order to obtain a more accurate account of this widely reported association. The prevalence rates of current smoking for adolescents aged 12-15 years and those aged 16-18 years were found comparable (i.e. approx. 4% and 24% respectively) to those reported in other Greek studies [2,31]. The frequency of current smokers was significantly higher among adolescents from low family socioeconomic background and with a smoking parent. These findings are consistent with previous data [30-32]. However, the present study could not detect any significant association between smoking status and adolescents' leisure time [31]. The measurement of leisure time applied here may account for this finding, since it did not include data regarding parental control and surveillance over adolescents' activities outside school and family. Cigarette smoking was associated with higher levels of emotional/behavioural problems and the association was not moderated after controlling for the effects of other covariates. Emotional symptoms, conduct problems and hyperactivity/inattention were all significantly associated with adolescents' current smoking, lending further support to existing literature [5-21,23-25]. This result also suggests that the association between smoking and emotional/behavioural problems is not confounded by significant predictors for both adolescents' smoking and emotional/behavioural problems, such as age, socioeconomic status and parental smoking.

The reported relationships underlie the fact that it is often the same individuals who engage in risk behaviours such as smoking and present emotional/behavioural difficulties, increasing the harmful effect of these behaviours on human health. The present findings highlight the importance of addressing adolescents' mental health problems in any effective effort for preventing or combating adolescents' cigarette smoking. Instead of dealing with smoking as a more or less normative risky behaviour during this critical period or focusing mainly on peer influences, adolescent health professionals should take into account the complex cluster of problems and needs which possibly burden adolescents and eventually remain unmet.

Certain limitations should be considered in understanding the results of the present study. The cross-sectional design of the study could not demonstrate causal directions between smoking and emotional/behavioural problems. Additionally, as is frequently observed in school-based surveys, there was a tendency for a higher response rate from girls compared with boys. It should be stressed, however, that the methodology of the European project, within which the present study was conducted, achieved a sufficient degree of representativeness to provide reference population values, as provided elsewhere [34]. Moreover, it is possible that factors other than the variables included in the statistical analyses are related to the associations between smoking and emotional/behavioural problems. For example, genetic factors, smoking habits during pregnancy, peers' smoking and personality variables (e.g. coping styles, affect regulation, risk-taking) could mediate or moderate the abovementioned relationship. Finally, tobacco use was not assessed through elaborated measures of smoking behaviour (tracking initiation age and frequency with a longitudinal design), which may have misclassified the smoking status with consequences on the observed associations.

## Conclusion

This study, along with previous research, supports the association between smoking and emotional/behavioural problems among adolescents. Addressing adolescents' needs regarding their emotional/behavioural health could be helpful in the development of effective anti-smoking strategies in school environment and elsewhere. Targeting vulnerable groups, such as socioeconomically disadvantaged adolescents or children with smoking family members should be combined with a thorough attempt to respond to concurrent emotional/behavioural problems in order to both promote a smoke-free lifestyle and enhance general wellbeing and functioning. Reversely, smoking in adolescence should be considered as a possible indicator for emotional/behavioural problems and thus, can help peers, parents and teachers be aware of adolescents at risk for problems such as emotional/behavioural difficulties which may be difficult to identify.

## Competing interests

The authors declare that they have no competing interests.

## Authors' contributions

GG, CT, CD, GK and VR participated in the preparation of the paper. YT had overall supervision of the study. All authors read and approved the final manuscript.

## Pre-publication history

The pre-publication history for this paper can be accessed here:

http://www.biomedcentral.com/1471-2458/10/57/prepub
